# Supplementary figures and images for: Immunophenotypic Analysis of Hairy Cell Leukemia (HCL) and Hairy Cell Leukemia-like (HCL-like) Disorders
Source: Cancers (Basel). 2022 Feb 18;14(4):1050. doi: 10.3390/cancers14041050 (PMC8870214; doi:10.3390/cancers14041050)

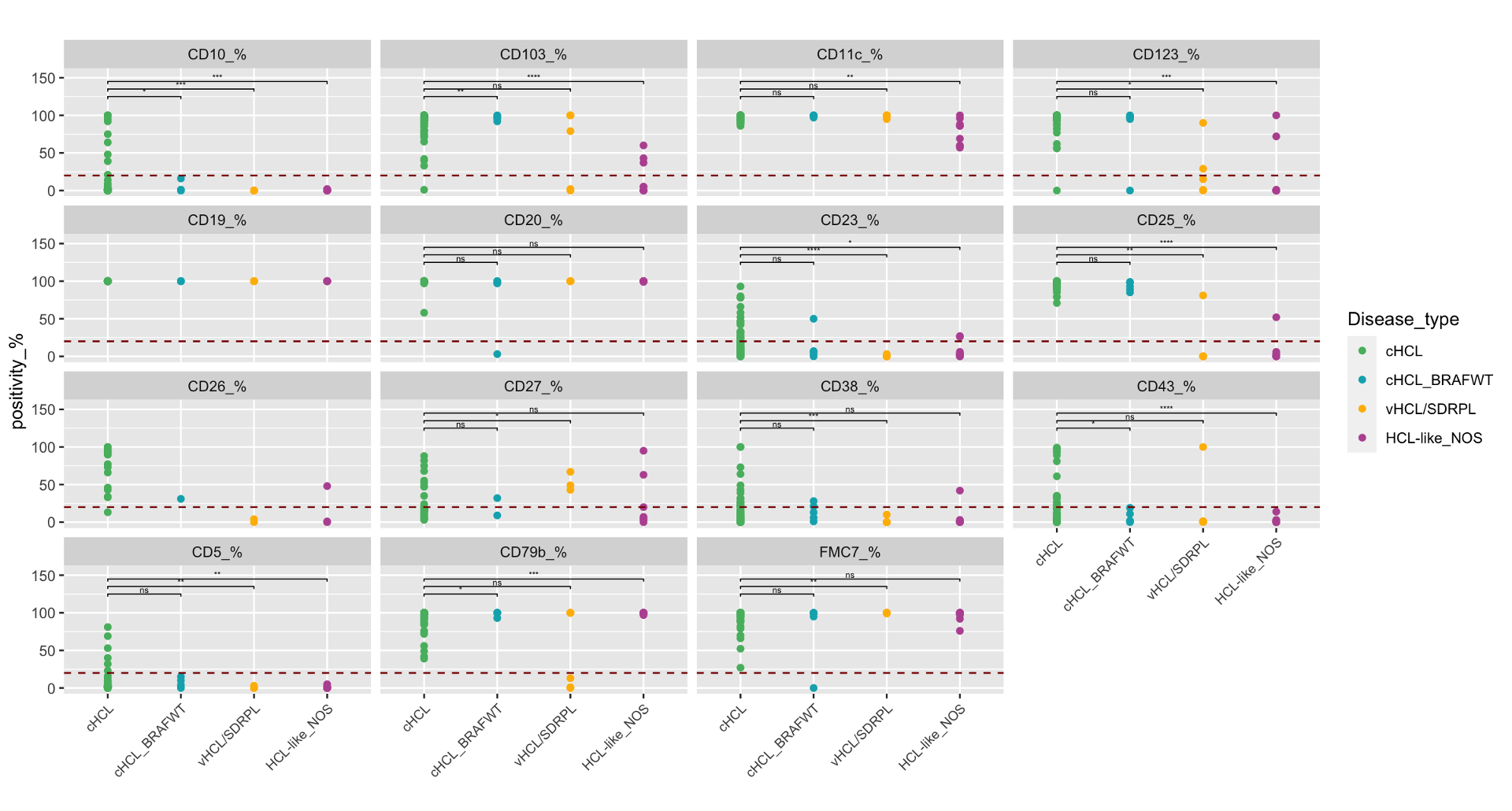

Supplement: Supplementary file 1 [file cancers-14-01050-s001.zip › cancers-1555997-supplementary/Figure S1 expression for each marker tested.png]

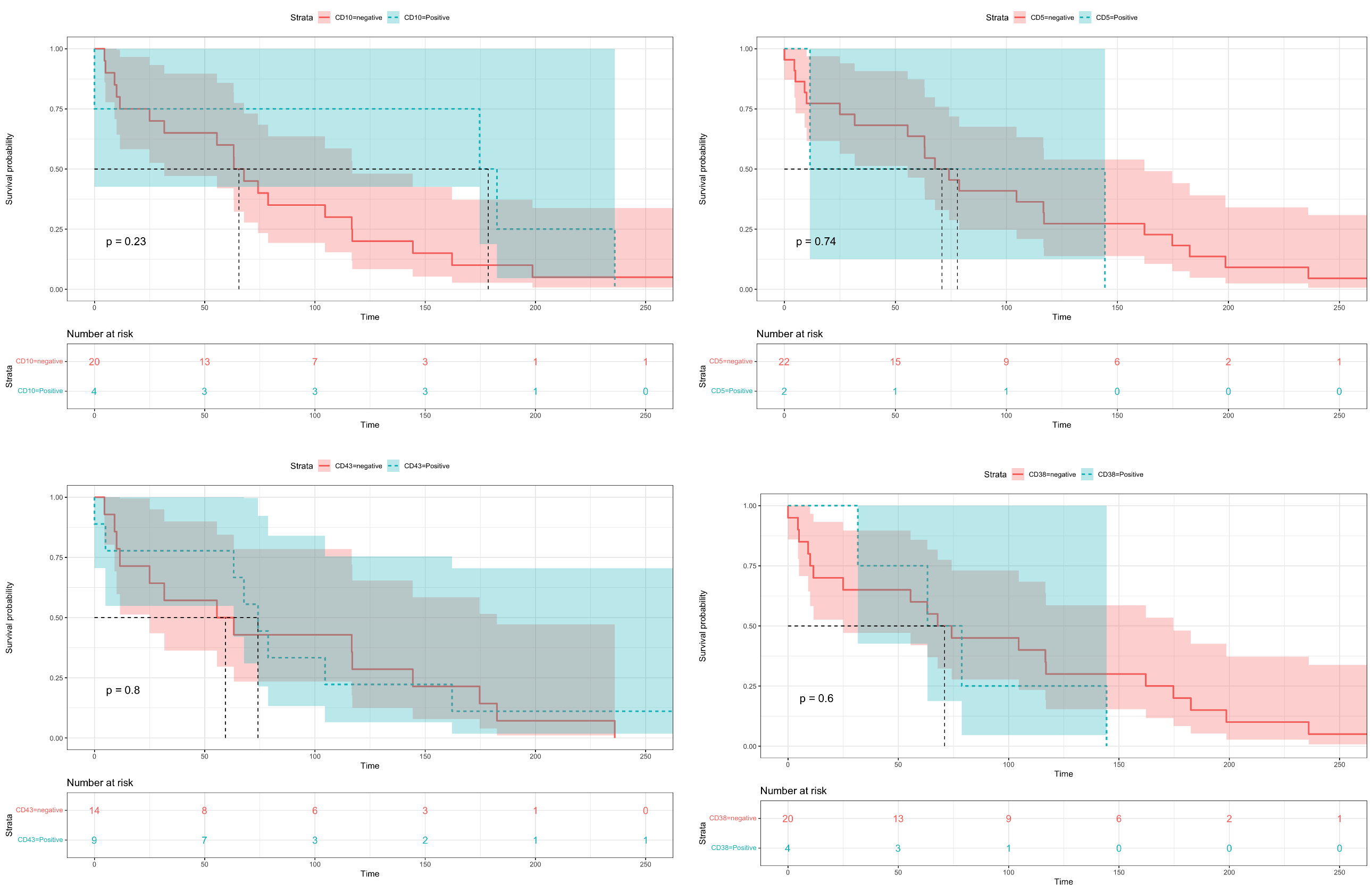

Supplement: Supplementary file 1 [file cancers-14-01050-s001.zip › cancers-1555997-supplementary/Figure S2 a TTNT according CD10, CD5, CD43, CD38.png]

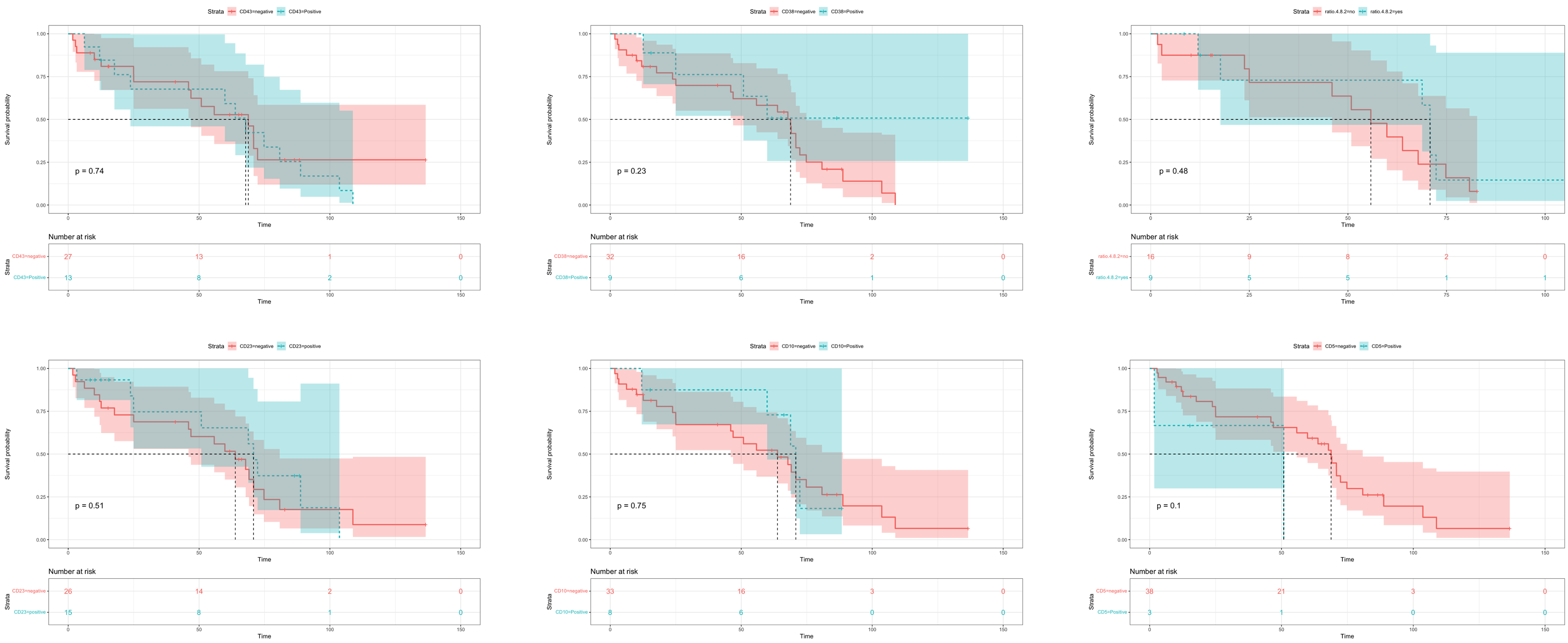

Supplement: Supplementary file 1 [file cancers-14-01050-s001.zip › cancers-1555997-supplementary/Figure S2 b PFS according to the expression CD23, CD4CD8, CD10, CD5, CD4....png]

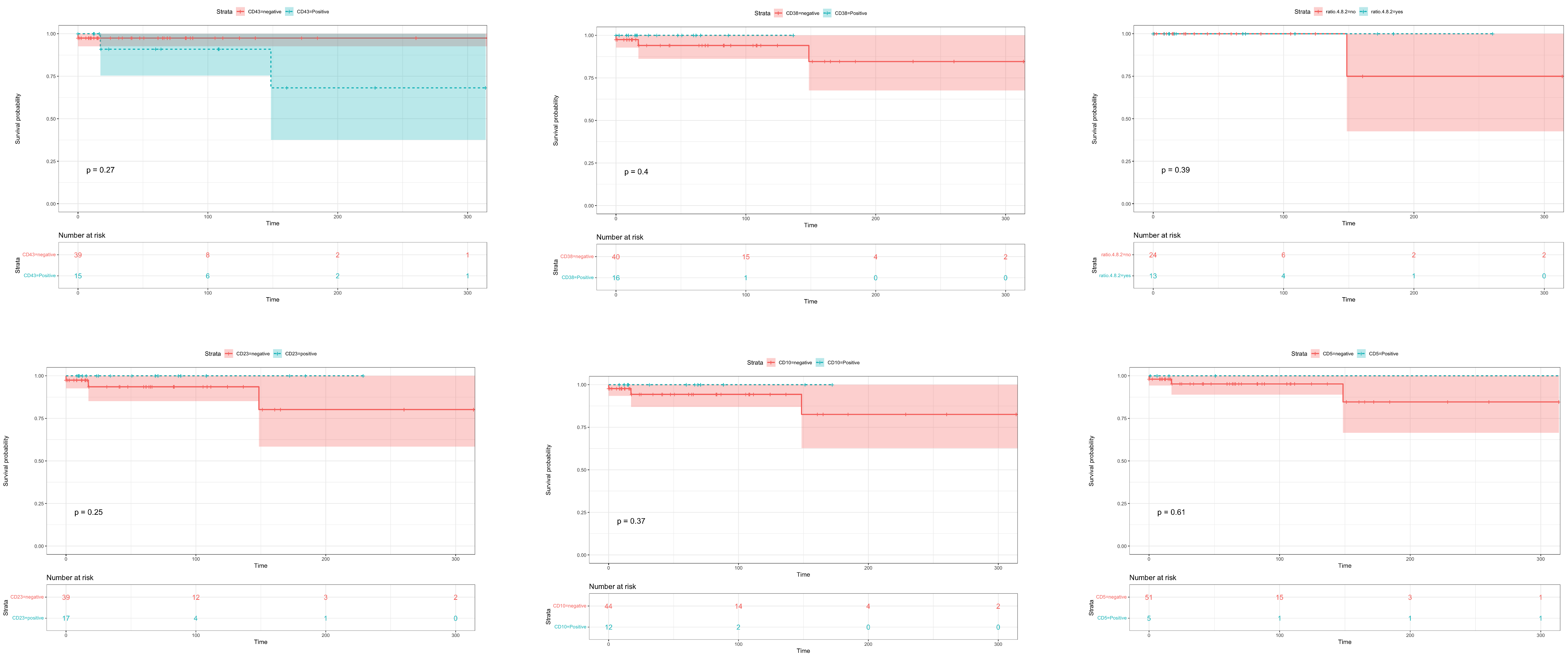

Supplement: Supplementary file 1 [file cancers-14-01050-s001.zip › cancers-1555997-supplementary/Figure S2 c OS according CD23, CD4CD8, CD10, CD5, CD43, CD38.png]

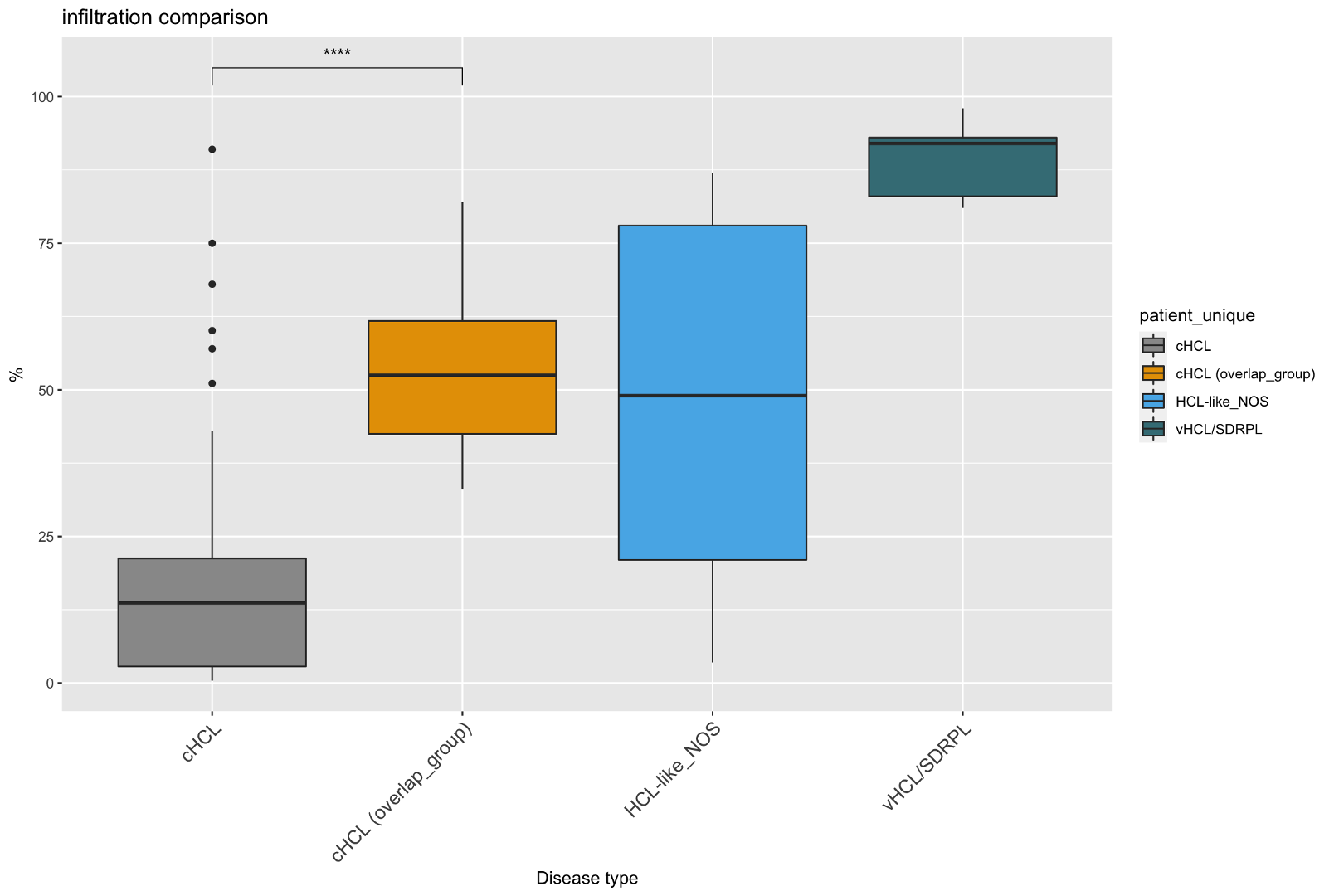

Supplement: Supplementary file 1 [file cancers-14-01050-s001.zip › cancers-1555997-supplementary/Figure S3 % of infiltration.png]

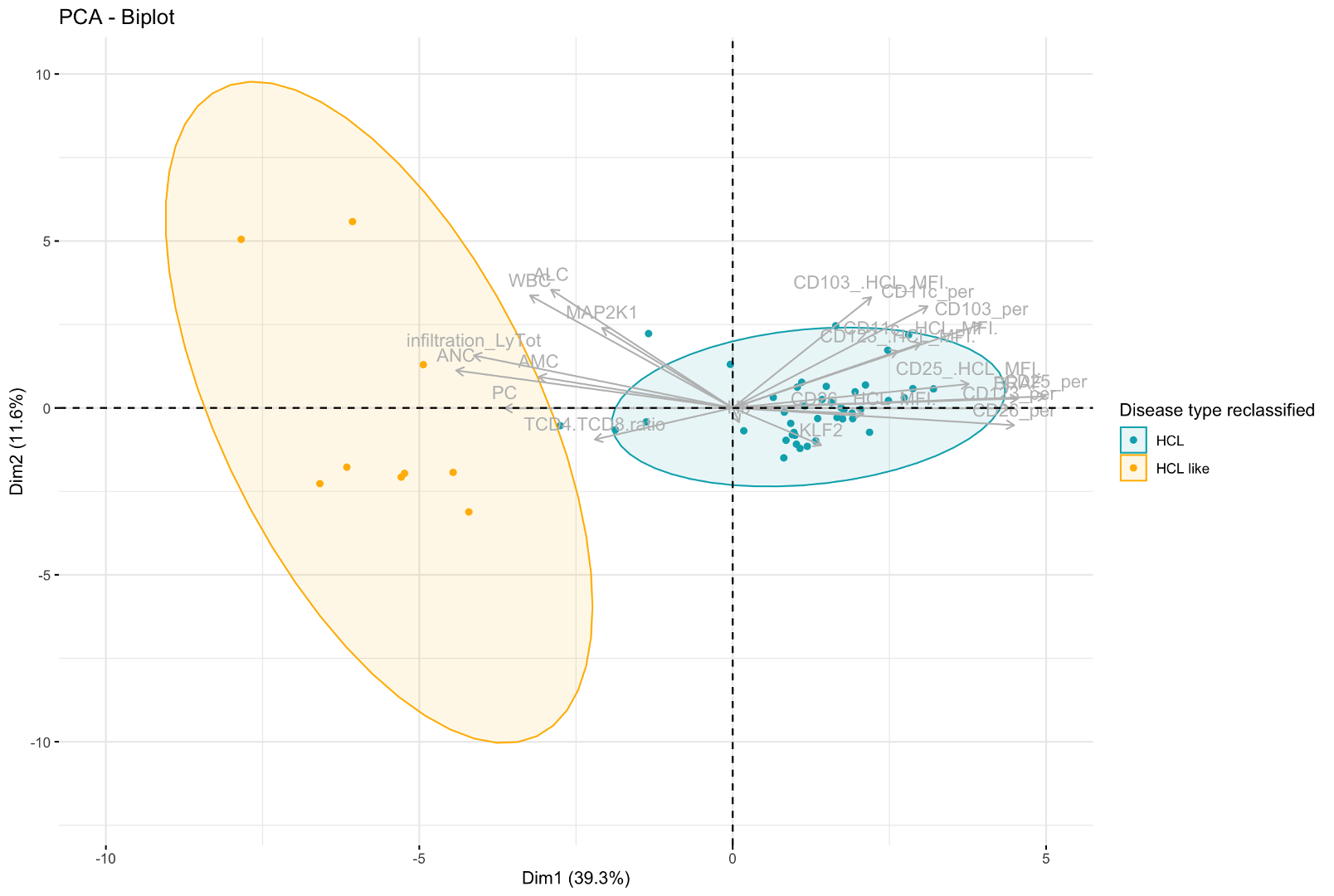

Supplement: Supplementary file 1 [file cancers-14-01050-s001.zip › cancers-1555997-supplementary/Figure S4 CPA.PNG]
